# Simulating Public Opinion: Comparing Distributional and Individual-Level Predictions from LLMs and Random Forests

**DOI:** 10.3390/e27090923

**Published:** 2025-09-02

**Authors:** Fernando Miranda, Pedro Paulo Balbi

**Affiliations:** 1 Programa de Pós-Graduação em Engenharia Elétrica e Computação, Universidade Presbiteriana Mackenzie, São Paulo 01302-000, SP, Brazil; 2 Faculdade de Computação e Informática, Universidade Presbiteriana Mackenzie, São Paulo 01302-000, SP, Brazil; pedrob@mackenzie.br

**Keywords:** LLM, random forests, Jensen–Shannon divergence, public opinion simulation, social sciences

## Abstract

Understanding and modeling the flow of information in human societies is essential for capturing phenomena such as polarization, opinion formation, and misinformation diffusion. Traditional agent-based models often rely on simplified behavioral rules that fail to capture the nuanced and context-sensitive nature of human decision-making. In this study, we explore the potential of Large Language Models (LLMs) as data-driven, high-fidelity agents capable of simulating individual opinions under varying informational conditions. Conditioning LLMs on real survey data from the 2020 American National Election Studies (ANES), we investigate their ability to predict individual-level responses across a spectrum of political and social issues in a zero-shot setting, without any training on the survey outcomes. Using Jensen–Shannon distance to quantify divergence in opinion distributions and F1-score to measure predictive accuracy, we compare LLM-generated simulations to those produced by a supervised Random Forest model. While performance at the individual level is comparable, LLMs consistently produce aggregate opinion distributions closer to the empirical ground truth. These findings suggest that LLMs offer a promising new method for simulating complex opinion dynamics and modeling the probabilistic structure of belief systems in computational social science.

## 1. Introduction

Survey research has long been a cornerstone of social science, political science, and policy-making, providing critical insights into public opinion, voting behavior, and social attitudes. However, traditional surveys face increasing challenges: they are costly, time-consuming, and burden respondents with long questionnaires, all while suffering from declining response rates and concerns about data reliability. These limitations motivate the exploration of computational methods that could complement or extend survey research, particularly through the use of artificial intelligence.

Large Language Models (LLMs) represent a promising avenue for this purpose. Trained on vast human-written corpora, LLMs capture not only grammatical structure and world knowledge but also complex patterns of human beliefs, biases, and associations [1,2]. Many recent studies focus on the consequences of these biases and how to mitigate them [3,4]. On the other hand, recent studies have shown that, when properly conditioned, LLMs can simulate nuanced group-level opinions by leveraging these same biases they encode [5,6]. This has led to the emergence of “silicon sampling” methodologies, where LLMs are employed as synthetic survey respondents to replicate human-like patterns of political and social beliefs.

Silicon sampling [6] refers to the idea of conditioning language models on externally sourced demographic or attitudinal profiles—such as those from the ANES—in order to simulate responses from synthetic personas that mirror real population distributions. The goal is to correct for the mismatched marginal distributions inherent to LLM training data, allowing researchers to generate opinion estimates for more representative subpopulations. While promising, this approach carries theoretical limitations. First, the technique assumes that the LLM’s internal conditional distributions P(opinion|backstory) are sufficiently aligned with those of real humans. Second, it presumes that LLM-generated responses conditioned on these profiles generalize beyond the temporal, cultural, or linguistic context of the model’s training data. These limitations highlight the need for validation studies—like ours—that test LLM behavior at both the aggregate and individual levels, across a variety of background configurations and opinion domains.

Despite this progress, critical questions remain about the granularity at which LLMs can simulate opinions—particularly whether they can accurately replicate individual-level responses, how large these LLMs need to be, and how different types of background information affect their performance.

In this study, we address these questions by proposing a framework for simulating public opinion using LLMs conditioned on structured survey data. Specifically, we use the 2020 American National Election Studies (ANES) dataset to build “backstories” for individual respondents, categorizing their attributes into three distinct groups: *Demographic*, *Attitudinal and Political Orientation*, and *Moral and Social Values*. By selectively providing the LLM with different categories of variables, we investigate how the amount and type of background information impacts its ability to simulate individual survey responses across a range of political and social topics.

Our research seeks to answer the following questions:

1. How accurately can an LLM (Gemma3 12B and Qwen2.5 14B) simulate individual responses to diverse opinion questions based on structured ANES backstory data?
2. How does the type of information provided (Demographic, Attitudinal, Moral) influence the simulation accuracy? Which combinations of variable types are most effective?
3. How does the LLM’s performance compare to a standard machine learning classifier (Random Forest) trained on the same data and task?
4. How does the LLM’s ability to select relevant variables within a pool affect prediction accuracy?
5. How does simulation accuracy vary across different socio-political topics?

To benchmark the performance, we compare the LLM’s predictions against real survey responses using the F1-score and Cramer’s V for individual-level accuracy and Jensen–Shannon distance (JSD) for distributional similarity. Notably, while the LLM performs comparably to Random Forests on individual-level F1-scores, it outperforms them in capturing the distributional patterns of opinions across most topics. This suggests that even moderately sized LLMs, like Gemma3 12B, can serve as powerful tools for cost-effective and scalable simulations of public opinion at a collective level.

Our main contributions are as follows:

- We propose a new framework for conditioning LLMs on structured backstory variables from public survey data to simulate individual and collective opinions.
- We systematically analyze the impact of different types and combinations of background information on simulation accuracy.
- We directly compare LLM performance against traditional machine learning methods on the same survey prediction tasks.
- We demonstrate the viability of LLMs as synthetic populations for simulating public opinion distributions, opening new avenues for low-cost and scalable survey research.

The paper proceeds by first reviewing related work on LLMs and opinion simulation, followed by a detailed description of our methodology and experimental design. We then present each simulation in detail before discussing the main results, limitations, and future directions.

## 2. Related Work

LLMs have demonstrated remarkable capabilities in generating coherent and contextually appropriate text. These models, trained on vast amounts of data, can simulate human-like responses, making them valuable tools in various applications, including synthetic surveying and social network simulations.

Recent research has increasingly explored the potential of LLMs to simulate aspects of human behavior. Several studies examine how LLMs reflect human cognitive biases, replicate patterns of human error in reasoning, or capture nuanced differences across demographic groups [7,8,9]. While much of this work focuses on evaluating LLMs’ ability to mimic human responses at an aggregate or cognitive task level, fewer studies have addressed the challenge of simulating individual-level opinion patterns based on structured background information. This section reviews some works that are closely related to our own, examining their behavior and limitations. We organize these works into three categories—Persona Simulation, LLMs in Social Science, and Virtual Surveys—while acknowledging the significant overlap between them.

### 2.1. Persona Simulation

Recent work has explored using LLMs to create virtual agents or characters with coherent personalities and behaviors. For example, ref. [10] introduced Generative Agents: language-model driven software agents with memory, planning, and reflection components that wake up, cook breakfast, head to work, notice news, form opinions, and coordinate events in a simulated town. In evaluation these agents exhibited believable individual and emergent social behaviors (e.g., planning a community party via invitation chains), demonstrating the potential of LLMs to simulate not only isolated responses but rich social interactions.

Other work has focused on endowing LLMs with more detailed character traits. In [11] the authors trained a “CharacterBot” on the collected writings of Lu Xun (a Chinese author) to capture his worldview and ideology. Their multi-task fine-tuning yields outputs that better preserve linguistic style and ideological perspective than simpler methods. Similarly, ref. [12] construct the LifeChoice benchmark from novels to test if LLMs can predict characters’ decisions; they find state-of-the-art LLMs achieve promising accuracy, yet substantial room for improvement remains.

In human–computer interaction, ref. [13] built PersonaFlow, an LLM-based system that simulates multiple expert personas (e.g., chemist, political scientist) during a brainstorming session. They report that interacting with several simulated experts improves the relevance and creativity of ideas, though it raises concerns about over-reliance and biases. Across these studies, a common limitation is that LLM-generated personas can oversimplify or stereotype real human variability. In [14] the authors noted that LLM-based demographic simulations often produce “caricatures”—excessively stereotyped or homogenized personas—and they propose metrics to quantify lack of individuation or exaggeration in model outputs. In general, current persona models require extensive curated data or prompt engineering and may fail to generalize beyond the studied characters.

### 2.2. LLMs in Social Science Experiments

In [15] the authors introduce the concept of *Turing Experiments (TEs)*, a novel method to evaluate the zero-shot simulation capabilities of LLMs. In TEs, models are prompted to generate text transcripts simulating human responses across various experimental settings, offering insight into which human behaviors LLMs can replicate. The authors conducted four TEs across distinct domains: behavioral economics, psycholinguistics, social psychology, and collective intelligence. Their results show that LLMs can capture meaningful human-like behavioral patterns, often with higher fidelity in larger models. However, they also highlight important challenges, including potential contamination from training data, the risk of prompt-induced biases, and ethical concerns in simulating sensitive aspects of human behavior. Overall, TEs provide a valuable tool for exploring how LLMs internalize and reflect complex social and cognitive behaviors, while underscoring the need for careful experimental design and interpretation.

Other work has adapted LLMs to field settings: In [16] the authors created an automated pipeline to extract information from 319 published field experiments (economics/social science) and prompt GPT-4 to choose outcome predictions; they achieve roughly 78% accuracy overall, though performance is highly uneven (e.g., worse for studies on gender or non-Western contexts). Finally, ref. [17] introduce SocioVerse, a synthetic world built from 10 million “user profiles” and LLM-driven agents. In domains of politics, news, and economics, SocioVerse’s agents collectively reproduce population dynamics (e.g., election outcomes, public opinion shifts) while preserving diversity of attitudes.

These modeling efforts suggest that LLMs offer a promising new tool for social science: they can accelerate hypothesis testing and policy exploration without real subjects (reducing cost and ethical constraints) [18]. However, existing systems face clear limitations. LLMs ultimately rely on patterns in their training data, so their “inferences” may reflect historical and linguistic biases. They tend to underperform out-of-distribution or minority scenarios [19] and can produce overly confident or uniform answers compared to diverse human behavior [15]. Experts therefore caution that LLM-based simulations should be used cautiously and primarily for exploratory research, with repeated runs and careful variability analysis.

### 2.3. Virtual Surveys and Synthetic Respondents

Another emerging line of work treats LLMs as virtual survey participants, generating synthetic responses under given demographic profiles, which closely relates to our own work.

In [5] the authors introduce a framework for analyzing whose opinions LLMs reflect when responding to subjective questions across various topics, ranging from religion to science. The authors create OpinionQA, a dataset based on high-quality public opinion polls that allows for evaluating how LLM opinions align with those of 60 U.S. demographic groups. In their primary analysis, the authors directly asked the LLMs the survey questions without any demographic-specific prompting or roleplaying instructions. This revealed a substantial misalignment between views expressed by current LLMs and those of U.S. demographic groups. In their “steerability” experiments, most models do tend to become better-aligned with a specific group when prompted to behave like it; however, these improvements are modest. Here, we should note that the authors focused on demographic traits to condition the models. In contrast, our work goes further by incorporating attitudinal and moral dimensions into the respondent profiles. As we will demonstrate, this richer modeling helps explain why prior work observed only limited steerability: demographic factors alone are often insufficient to capture the full complexity of human opinions.

In [6] the authors introduce the concept of silicon sampling, proposing that LLMs like GPT-3 can serve as effective surrogates for human respondents in social science research. Their key idea is that “algorithmic bias” in LLMs is not a flaw but reflects fine-grained, demographically correlated patterns that can be systematically leveraged. In their study, the authors condition GPT-3 on thousands of real socio-demographic backstories drawn from large U.S. surveys, generating “silicon individuals” to simulate tasks such as vote choice prediction and answering closed-ended survey questions. While their work provides strong evidence that GPT-3 can simulate aggregate human responses when conditioned on demographic variables, it primarily focuses on traits such as race, gender, age, and political affiliation.

In [20] the authors probed ChatGPT-4 by assigning it demographic profiles from the World Values Survey and ANES. GPT-4 reproduces many cultural differences and “in-sample” response patterns across U.S. and Chinese subgroups, and even produced correct forecasts for the 2024 U.S. presidential election. However, it shows limitations on value-sensitive questions and an overall tendency to homogenize responses: subtle prompt phrasing or demographic details can substantially affect answers. In [21] the authors conducted a multi-country test by prompting GPT variants, Llama2, etc., to answer items from the European Social Survey on politics and democracy. They found that with a few relevant examples (“few shot prompts”) LLMs generate highly realistic answers, but in zero-shot mode (no examples) performance degrades markedly. Collectively these studies find that LLMs can simulate group-level opinion distributions, but major challenges and questions remains. In the next sections, we describe our approach to addressing the questions posed in the Introduction, developing a persona construction strategy that moves beyond demographic attributes to incorporate attitudinal and social values.

## 3. Materials and Methods

The ANES (American National Election Studies) is a respected research organization founded in 1948 that conducts comprehensive pre- and post-election surveys in the United States. These surveys collect extensive data on voter behavior, political attitudes, and public opinion, creating valuable datasets for social science researchers. In the next section we describe in detail the subset of the ANES 2020 dataset that we used in our work.

### 3.1. ANES 2020 Dataset

The ANES 2020 dataset contemplates interviews with respondents between 18 August 2020, and 3 November 2020, being the primary data source for comprehending the American public opinion. The dataset includes demographic, social, and behavioral information, including responses to presidential votes and political surveys. The dataset also features re-interviews with 2016 ANES respondents, and has samples from the 2020 pre-election and post-election periods.

In our work we focus exclusively on respondents from the 2020 pre-election sample, excluding the ANES 2016 respondents as well as post-election samples. We then select two groups of variables: the first we will denote by *backstory variables*, and the second simply by *topics*. The backstory variables are the set of 25 variables that can be used to compose the “persona profile” of our virtual person. The topics are a set of 10 questions that this virtual person will answer when roleplayed by the LLM. We also group the backstory variables into three distinct groups: Demographic (D), Attitudinal and Political Orientation (A), and Moral and Social Values (M). For brevity, we often refer to the last two groups simply as “Attitudinal” and “Moral” variables, respectively.

Table 1, Table 2 and Table 3 present the response options available to survey participants. The interpretation of the Demographic and Attitudinal variables is self-explanatory. Among the Social Values variables, the “Preferred Child Trait” item asks respondents to select the child characteristic they value most from the provided options. The “Birthright Citizenship End” variable measures how strongly respondents favor or oppose ending automatic citizenship for children of unauthorized immigrants. Variables prefixed with “Discrimination” gauge respondents’ perceptions regarding the prevalence of specific types of discrimination at the time of the survey. The “Historic Racism Impact” item assesses respondents’ agreement with the statement that generations of slavery and discrimination have created conditions that make life more difficult for Black Americans. Table 4 shows the topics we are interested in.

For the topics *Climate Change* and *Current Economy* (marked with “*”), the original survey presented 5 choices. We remapped them in the following way: for *Climate Change*, options “A little” and “A moderate amount” were collapsed into “A little”, and options “A lot” and “A great deal“ were collapsed into “A lot”. For *Current Economy*, options “Very Good” and “Good” were collapsed into “Good”, and options “Bad” and “Very Bad“ were collapsed into “Bad”. This improves interpretability and ensures sufficient sample sizes across response categories while retaining the substantive distinctions between low and high concern or between positive and negative evaluations [22]. All other topics are presented exactly as they appear in the survey.

### 3.2. Data Preparation and Model Selection

Each topic in our study is treated as an independent modeling task. For a given experiment, the input dataset consists of a specific set of backstory variables along with the specific topic variable as the prediction target. Each row of the dataset corresponds to a single survey respondent, fully characterized by their backstory and topic-specific response.

**Figure 1:**
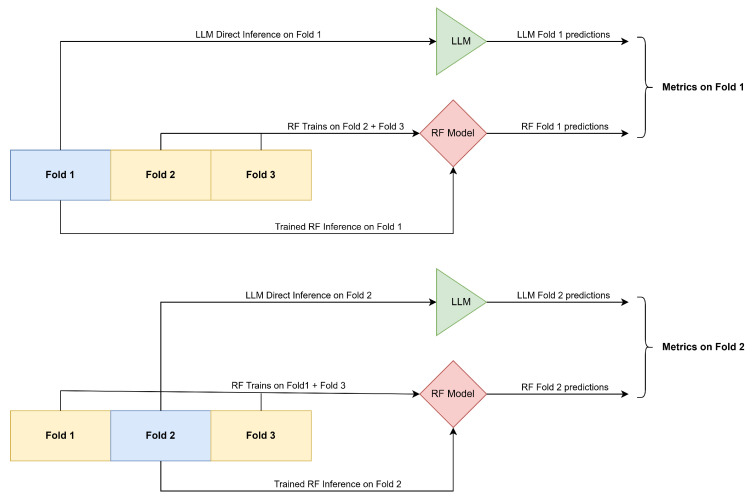
Data splitting schema: In the first split, the blue shaded rectangle (Fold 1) represents the validation fold (“prediction targets”). For an LLM, this is a direct inference on Fold 1 via backstory variables. For the RF, the model is first trained on Folds 2 and 3, then predictions are made on Fold 1. Finally, we have predictions from both models for the same set of respondents from Fold 1, and metrics for this fold are calculated. The same logic is shown for Split 2 (Split 3 is not shown for brevity). The metric reported is the average across the three validation folds.

Since we aim to compare the performance of LLMs against a traditional Random Forest (RF) classifier, it is essential that both models are evaluated on the same validation sets. To ensure fairness and maximize robustness, we adopt a 3-fold cross-validation strategy: in each fold, approximately 33% of the data is held out for validation, with the remaining 67% used for training (only for the RF). This procedure guarantees that all data points are used for validation exactly once, reducing variance and minimizing potential biases. All reported performance metrics are computed as the simple average across the three validation folds. Figure 1 illustrates this schema.

In the early stages of this work, we conducted preliminary comparisons between Random Forests, Linear Classifiers, and Gradient Boosting Machines (all from scikit-learn). Among these, RF consistently outperformed the linear models and outperformed the gradient boosting model on most topics. Although gradient boosting can be highly effective when carefully tuned, we found its performance to be less stable across topics under a fixed parameter setup. Given our goal of comparing an LLM to a robust, interpretable, and fair out-of-the-box benchmark, Random Forests were a natural choice. They are well-suited to datasets with predominantly ordinal and categorical variables, require minimal preprocessing, and are widely used in computational social science. Their robustness to overfitting and strong default performance made them ideal for ensuring consistency and comparability across experiments.

We evaluated two Large Language Models: Gemma3 12B [23] and Qwen2.5 14B [24]. Both models represent high-performing, state-of-the-art models among smaller-scale LLMs, making them well-suited for this research. While the main body of our article focuses on results obtained using Gemma3—our primary LLM throughout the study—we also include a comparison with Qwen2.5 to explore the external validity of our findings across models with different alignment and training origins. Qwen2.5 is particularly relevant as it reflects a non-Western alignment paradigm, offering insight into how cultural and normative differences may influence LLM behavior in social science simulations. For clarity and conciseness, we present the key summary tables for Qwen2.5’s performance in Experiments 2 and 3 in Appendix B. A full duplication of all tables and heatmaps was omitted, for this model, as it would be largely redundant and would not alter the study’s core conclusions. For the complete Qwen2.5’s data results, we refer to our code repository.

Finally, it is important to highlight again that **no fine-tuning or training** is performed on the LLMs; it uses only the backstory information at inference time. Thus, the training data is utilized exclusively by the RF model. Lastly, respondents with missing data in any relevant fields were excluded from the analysis to maintain consistency across models.

### 3.3. Evaluation Metrics Statistical Analysis

To quantify the statistical uncertainty of our reported performance metrics (F1-score, JSD, Cramér’s V) and the differences between models or experimental conditions, we employed a non-parametric bootstrap procedure. For each primary metric averaged across the 3 cross-validation folds, B = 1000 bootstrap iterations were performed. In each iteration, (prediction, true label) pairs were resampled with replacement from each fold’s original validation set (maintaining original fold sample sizes). The metric was calculated for these resampled data within each fold, and these three fold-level metrics were then averaged to produce one bootstrap estimate of the mean cross-validated metric. This process yielded a distribution of 1000 bootstrap estimates for each reported mean metric or mean difference. From these distributions, 95% confidence intervals (CIs) were derived using the percentile method (i.e., the 2.5th and 97.5th percentiles). Differences or gains were considered statistically significant at the *p* < 0.05 level if their corresponding 95% CI did not include zero. In the main text tables we mark the significant values with an asterisk (*). The full CI data can be found in the Appendix B.

## 4. Results

### 4.1. Experimental Sequence

**Figure 2:**
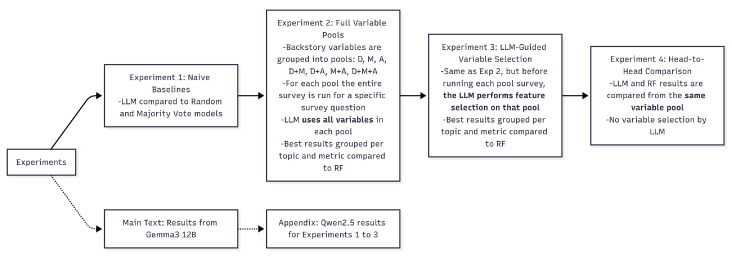
Experimental sequence: The diagram shows how experiments build upon each other.

In the next sections we conduct four sequential experiments to evaluate and compare model performance. We begin by establishing a LLM baseline against naive models, using limited profile information (Exp 1). We then examine how access to more comprehensive variable pools affects model behavior (Exp 2). In the third experiment, we evaluate the model’s capacity for autonomous feature selection (Exp 3). Finally, we present the head-to-head comparison under identical conditions to isolate each model’s distinct capabilities (Exp 4). Figure 2 illustrates the sequence.

### 4.2. Experiment 1—Random and Constant Model Baselines

Before evaluating model accuracy or variable grouping effects, we establish a performance baseline using two naive models: a *Random Model* and a *Constant Model*. These serve as essential lower bounds for interpreting the behavior of more advanced models. All experimental results presented in the main body of the article refer to Google’s Gemma3 12B model, which, for brevity, we refer to throughout as “the LLM.” Results for Qwen2.5 are included in the Appendix B for comparison.

The Random Model simulates a respondent who selects answers uniformly at random, representing a performance floor with no reliance on input features. The Constant Model always predicts the most frequent class from the training data, reflecting population-level bias but ignoring respondent variation. This is especially relevant in imbalanced datasets, where majority-class predictions can appear deceptively strong.

These baselines help determine whether the LLM captures meaningful patterns or merely mimics simple heuristics. To keep the benchmark experiment as simple and interpretable as possible, we constrain the LLM to select only **one backstory variable from each of the three groups**. For each topic, the model selects the most predictive variable per group using its internal knowledge. These are then used to construct the virtual respondent’s prompt. This setup provides a minimal yet structured input, enabling consistent evaluation across topics. Prompt templates for this experiment are included in the Appendix A. We also note that for all LLM experiments, the *temperature* parameter was held fixed at a value of 0.3. A low temperature of 0.3 was chosen to reduce randomness and encourage the model to produce the most probable, deterministic response based on the provided persona. Previous work like [6] found the temperature parameter to produce stable results, but since our work uses a different setting, we plan to study temperature effects more deeply in future works.

As shown in Table 5, the LLM consistently and substantially outperforms both the Random and Constant baseline models across all 10 survey topics and all three evaluation metrics (F1-score, Jensen–Shannon Distance (JSD), and Cramér’s V). For instance, on the “Climate Change” topic, the LLM achieved an F1-score of 0.59, compared to 0.36 for the Random model and 0.41 for the Constant model. Similarly, its JSD of 0.21 was markedly better (lower) than the Random model’s 0.29 and the Constant model’s 0.51, indicating superior distributional alignment. The Cramér’s V also showed a clear advantage for the LLM (e.g., 0.41 for “Climate Change” vs. 0.04 for Random; Constant model has no basis for Cramér’s V calculation as it does not use input features). The 95% confidence intervals for Experiment 1 results can be found in the Appendix B.

This pattern of LLM superiority over the naive baselines holds true across all topics. For example, for “Gay Marriage”, the LLM’s F1-score (0.68) and JSD (0.10) far exceeded those of the Random (F1: 0.38, JSD: 0.33) and Constant (F1: 0.59, JSD: 0.40) models. However, we point out two statistically significant exceptions: First, in the “Drug Addiction” topic, where the Constant model’s F1-score was slightly better (+0.02) due to class imbalance. In this case the LLM demonstrated a significantly better JSD (0.20 vs. 0.41), showcasing its superior ability to capture more than just the majority class. The second, in the “Refugee Allowing” topic, where the JS for the Random model was 0.15 better. Here, the LLM was better at both F1-score and Cramér’s V, suggesting that the variable selection made by the LLM for this specific topic was not good enough. This is confirmed in the next experiments, where the JS for the topic “Refugee Allowing” is significantly better than the one achieved by the Random model.

These results affirm that even with a highly constrained set of input variables, the LLM captures meaningful predictive patterns from the backstory information, performing well above chance and simple heuristics. This validates its potential for opinion simulation and sets the stage for exploring its capabilities with more comprehensive informational inputs in subsequent experiments.

### 4.3. Experiment 2—Impact of Backstory Variable Categories on Simulation Accuracy

In Experiment 1 we established a performance baseline where the LLM was constrained to utilize a minimal set of information: one self-selected predictor variable from each of the three primary backstory categories, totaling at most three variables. Building upon this, in this section we investigate the potential performance gains and the differential impact achieved when the LLM has access to all variables in each of the seven distinct combinations of these “variable pools”.

The 25 backstory variables are grouped into three categories (Table 1, Table 2 and Table 3), resulting in seven combinations or “variable pools”. For each *topic + variable pool*, the LLM is explicitly instructed to use all variables from the pool to make predictions. The prompt templates can be found in the Appendix A.

We also trained and evaluated RF classifiers on the exact same data. A separate RF classifier is trained for each topic + variable pool combination, using only the backstory variables from the given pool as input features and the corresponding topic survey response as the target. Both inputs and targets are encoded as integers. The training parameters for the RF classifiers are held constant across all experiments and are detailed in the Appendix B.

In Table 6, Table 7, Table 8 and Table 9 we summarize the best performing models across variable pools, for each topic. Table 6 and Table 7 detail the peak F1-score, JSD, and Cramér’s V achieved by the Gemma3 12B LLM and the RF model, respectively, along with the specific variable pool yielding this optimal performance for each topic and metric. A noteworthy initial observation is that these optimal variable pools frequently differed between the LLM and RF for identical tasks, suggesting distinct information utilization strategies by each model. Table 8 quantifies the LLM’s performance gains in this experiment relative to the more constrained Experiment 1 baseline, while Table 9 directly compares the peak LLM performance against the peak RF performance. Pool names are abbreviated for space.

Analysis of the LLM’s optimal configurations (Table 6) reveals that variable pools incorporating Attitudinal (A) or Moral (M) attributes, or their combination (A+M), frequently yielded the best results across most topics and metrics. For instance, on the “Climate Change” topic, the A+M pool provided the LLM’s highest F1-score (0.70) and best JSD (0.15). As detailed in Table 8, these scores represented substantial F1 and JSD gains of 0.11 and 0.07, respectively, over the Experiment 1 baseline, indicating that richer psychological information significantly enhanced simulation accuracy for this topic. Conversely, for “Gay Marriage”, while the A pool yielded the LLM’s best F1-score (0.66), this was a slight decrease from the Experiment 1 baseline, and the JSD also showed a decline, suggesting the simpler Experiment 1 setup was more effective for these specific metrics, though Cramér’s V did see a marginal improvement.

No single variable pool proved universally optimal. While A or M variables were prevalent in high-performing configurations, Demographic (D) variables tended to contribute most effectively in combination with other types, particularly for topics with inherent demographic components like “Gender Role”. For distributional similarity (JSD), the A pool demonstrated broad effectiveness, notably achieving the largest JSD gain over the baseline (+0.26) for “Refugee Allowing”. The magnitude of these gains over the Experiment 1 baseline varied considerably; substantial JSD improvements were also seen for “Health Insurance Policy” (+0.17 with D+A pool) and “Race Diversity” (+0.14 with A pool). While providing more extensive backstory information in Experiment 2 generally benefited LLM performance, especially for JSD, the Experiment 1 baseline occasionally remained competitive or superior, highlighting a nuanced interplay between question type, evaluation metric, and the most pertinent conditioning information.

A direct comparison between the peak LLM performance and peak RF performance, with each model utilizing its respective optimal variable pool (Table 9), reveals distinct capabilities. The LLM consistently demonstrated superior or equal ability in matching overall opinion distributions, achieving a statistically better JSD on six out of ten topics. Particularly notable JSD advantages for the LLM were observed for “Drug Addiction” (+0.18) and “Gender Role” (+0.18), underscoring its proficiency in capturing nuanced collective response patterns.

Regarding individual prediction accuracy (F1-score), the comparison was more balanced. The RF model often achieved a marginally higher peak F1-score, with a more pronounced advantage for “Gun Regulation” where its F1-score was 0.08 points higher than the LLM’s. Similarly, for associative strength (Cramér’s V), the RF model exhibited a clear superiority for “Gun Regulation” (−0.19) and “Race Diversity” (−0.17), while the LLM held a slight edge for “Drug Addiction” (+0.06), with minimal differences on other topics.

Experiment 2 demonstrates that while a RF classifier can achieve comparable or superior peak individual prediction accuracy (F1-score) and associative strength (Cramér’s V) on certain topics when operating with its optimal variable set, the Gemma3 12B LLM generally excels at replicating the distributional characteristics of survey responses (JSD). This distinction highlights their differing strengths: RF for robust predictive power on specific tasks, and LLMs for their nuanced capacity to simulate finer-grained collective opinion structures.

### 4.4. Experiment 3—Impact of LLM-Driven Feature Selection on Simulation Accuracy

Unlike Experiment 2, where the LLM utilized all variables within a given pool, in this experiment the LLM was first prompted to select only the variables it deemed most relevant for prediction from each topic and variable pool combination. Only these self-selected variables were then used to construct the respondent’s profile for simulation. This approach offers a practical advantage in reducing prompt token count, leading to more efficient inference. The RF model results from Experiment 2, where the RF used its optimal variable pool, serve as a benchmark for comparison.

Table 10 details the peak performance of the Gemma3 12B LLM in Experiment 3 across all topics and metrics when employing this feature selection strategy, along with the variable pool from which features were selected. A comparison of these results with the LLM’s performance in Experiment 2 (Table 6, where all variables in the optimal pool were used) reveals nuanced effects of feature selection. For F1-score and Cramér’s V, the differences were generally marginal across most topics, often close to zero. However, for JSD, LLM-driven feature selection yielded notable improvements in distributional alignment for several topics. For instance, JSD for “Income Inequality” and “Race Diversity” improved by 0.08 and 0.05, respectively. This suggests that by focusing on a curated set of variables, the LLM can sometimes enhance its ability to replicate collective opinion patterns.

Nevertheless, the efficacy of this internal feature selection was not uniform. A striking instance is the “Health Insurance Policy” topic, where JSD worsened by 0.11 when feature selection was employed. This decline coincided with the optimal pool for JSD shifting from D+A in Experiment 2 to A+M in Experiment 3, suggesting that the LLM’s feature selection may have deprioritized or omitted the crucial “has health insurance” demographic variable. This highlights a potential trade-off: while feature selection can enhance efficiency and, in some cases, improve JSD, it may occasionally overlook critical variables, particularly for topics with strong correlation to specific input features. The distribution of optimal variable pools also shifted in Experiment 3, with the D pool appearing more frequently as optimal for certain metrics compared to Experiment 2.

When comparing the peak LLM performance with feature selection against the peak RF performance from Experiment 2 (Table 11), the overall trends largely mirrored those observed in the Experiment 2 LLM vs. RF comparison. The LLM maintained its advantage in JSD for several topics, demonstrating strong distributional alignment. For example, for “Drug Addiction”, the LLM with feature selection achieved a JSD improvement of 0.23 over the peak RF performance, and for “Gender Role”, the improvement was 0.15.

Conversely, for individual prediction accuracy (F1-score), the RF model from Experiment 2 often held a slight edge or performed comparably to the LLM with feature selection. For “Gun Regulation” the RF outperformed the LLM by 0.07 in F1-score. Similarly, for associative strength (Cramér’s V), the RF demonstrated stronger performance for “Gun Regulation” (−0.18) and “Race Diversity” (−0.18), while the LLM showed an advantage for “Drug Addiction” (+0.09).

In essence, allowing the LLM to perform feature selection can lead to more efficient inference and, in some instances, can refine its ability to capture distributional patterns (JSD). However, its performance relative to a well-configured RF model remains largely consistent with the trends observed without this explicit LLM feature selection step: the LLM excels in distributional similarity while the RF often matches or slightly exceeds it in individual prediction accuracy on specific tasks.

### 4.5. Experiment 4: Head-to-Head Model Performance Across All Topic and Variable Pool Configurations

In both Experiments 2 and 3, we focused on identifying and comparing LLM and RF **peak model performances across variable pools**, which showed that optimal variable pools frequently differed between the LLM and RF for the same topic. In this section we present the direct comparison of these models under identical information conditions: for each topic we compare the performance under the same variable pool. To do this, we use the results from Experiment 2, since in this experiment the LLM utilizes all variables available within each pool, mirroring the RF’s input. This allows for a granular assessment of relative model strengths when the informational input is precisely matched.

**Figure 3:**
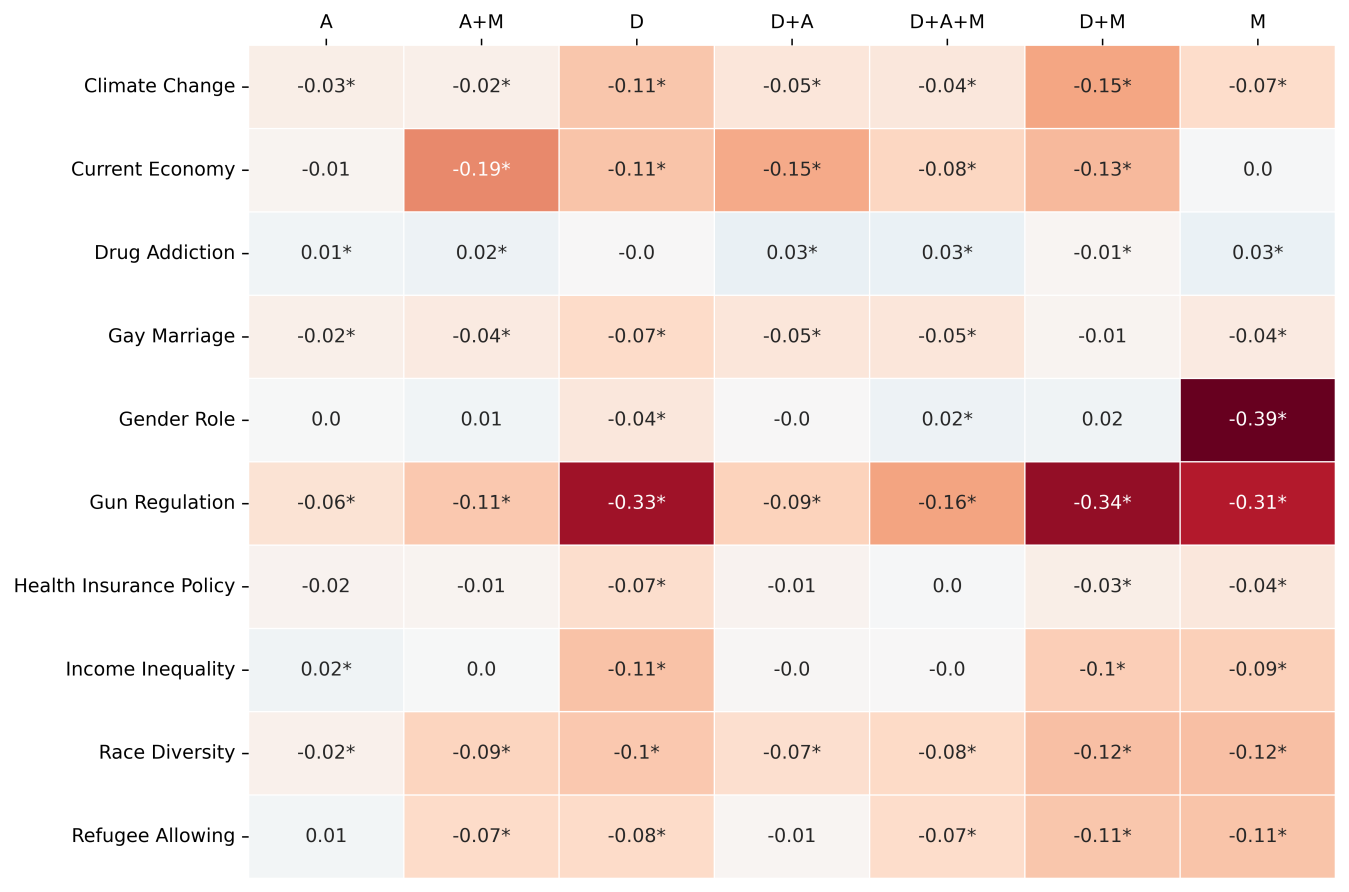
F1-score same pool comparison: [Gemma3 12B − RF]. The colors clearly show the performance pattern between the models. The * marks the statistical significance of the metric value.

**Figure 4:**
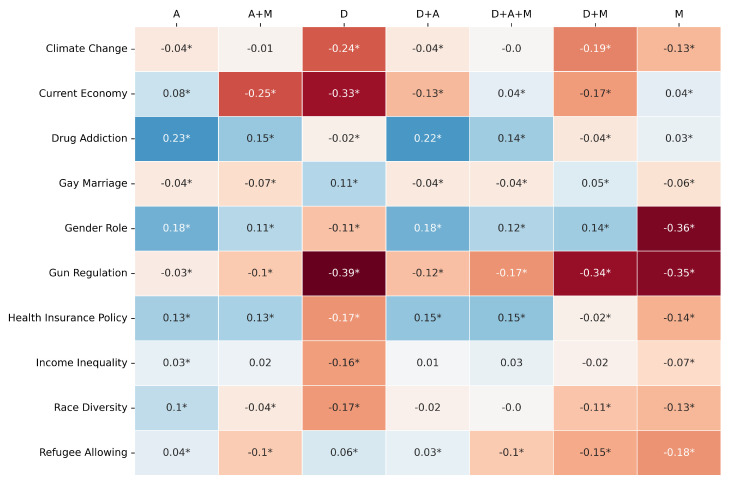
JS same pool comparison: [RF − Gemma3 12B]. The colors clearly show the performance pattern between the models. The * marks the statistical significance of the metric value.

**Figure 5:**
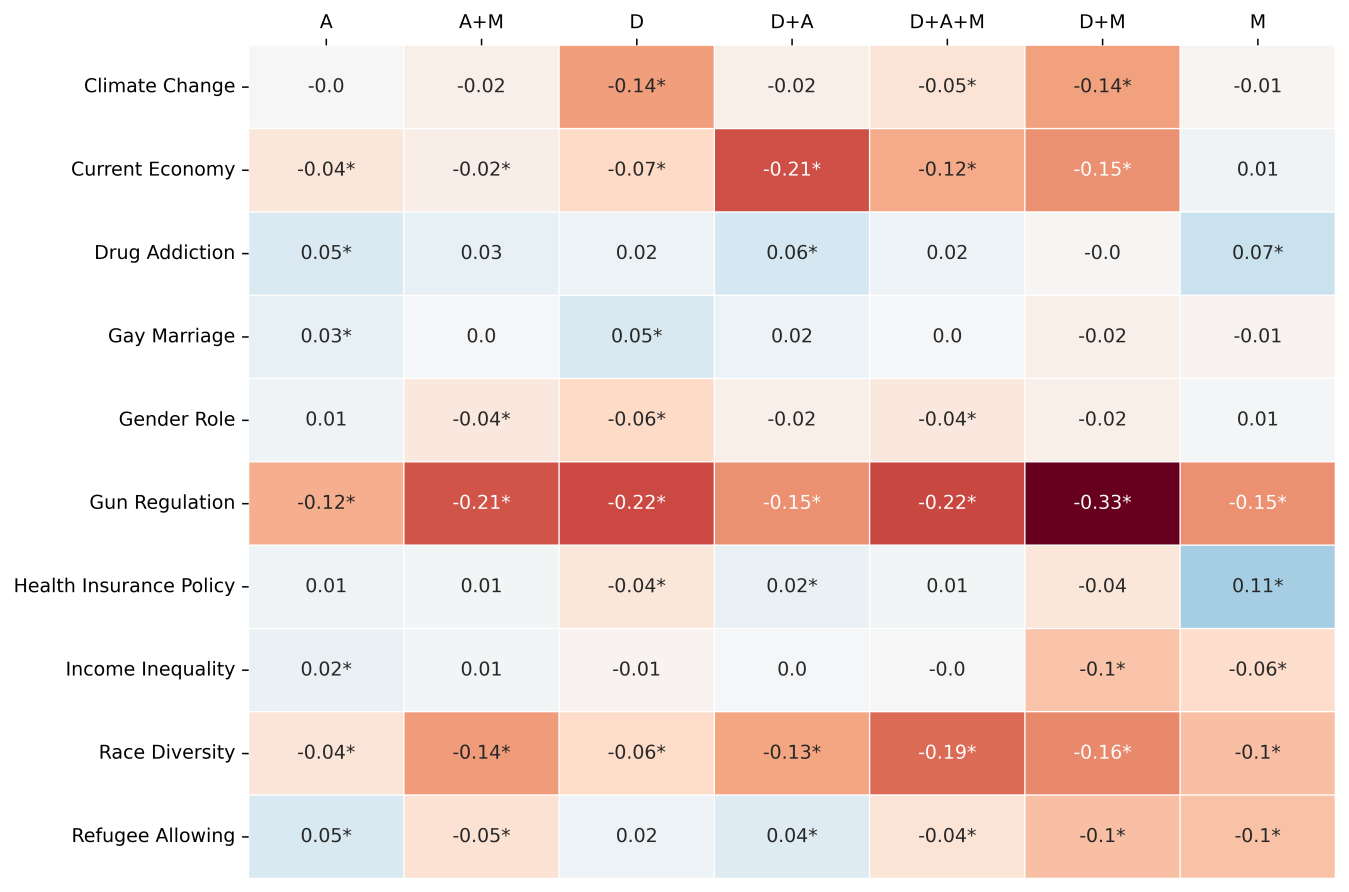
Cramér’s V same pool comparison: [Gemma3 12B − RF]. The colors clearly show the performance pattern between the models. The * marks the statistical significance of the metric value.

The comparative performance is primarily visualized through heatmaps (Figure 3, Figure 4 and Figure 5) depicting the difference between LLM and RF scores for F1-score, Jensen–Shannon Distance (JSD), and Cramér’s V, respectively. In these figures, rows represent the survey topics and columns denote the seven variable pools. Cell values quantify the performance gap (LLM score − RF score for F1-score and Cramér’s V; RF JSD − LLM JSD for JSD), with positive values (e.g., blue shades) indicating LLM superiority for that specific topic–pool combination. As in previous experiments, an asterisk (*) denotes a difference for which the 95% bootstrap confidence interval does not include zero, indicating statistical significance (*p* < 0.05).

An examination of these heatmaps reveals distinct patterns in the relative performance of the Gemma3 12B LLM and the RF when given identical inputs from each variable pool.

For F1-score (Figure 3), the RF model generally demonstrates an advantage or performs comparably to the LLM across most topic–pool configurations. This is evidenced by the prevalence of neutral (light/white) or orange/red shades. For instance, on “Gun Regulation”, the RF consistently outperforms the LLM across all variable pools, with particularly strong advantages when using the D (Diff: −0.33), D+M (Diff: −0.34), and M (Diff: −0.31) pools. Similarly, for “Current Economy“, the RF shows better F1-scores with pools like A+M (Diff: −0.19) and D+A (Diff: −0.15). The LLM achieves comparable or slightly better F1-scores in isolated cells, such as for “Drug Addiction” with the D+A pool (Diff: +0.03) or “Gender Role” with the D+A+M pool (Diff: +0.02), but these instances are less frequent. This suggests that for individual prediction accuracy under these matched input conditions, the RF model often has an edge.

The JSD comparison (Figure 4) presents a more favorable outcome for the LLM. The LLM frequently achieves superior distributional similarity across a variety of topics and pools. Notably, for “Drug Addiction”, the LLM shows strong JSD advantages with the A (Diff: +0.23) and D+A (Diff: +0.22) pools. For “Gender Role”, the LLM also performs well with the A (Diff: +0.18) and D+A (Diff: +0.18) pools. Similar LLM advantages in JSD are seen for “Health Insurance Policy” with A, A+M, D+A, and D+A+M pools. However, the RF can still achieve better JSD in some cases, particularly when the D pool is used for topics like “Climate Change” (Diff: −0.24) or “Current Economy” (Diff: −0.33), and notably for “Gun Regulation” with the D pool (Diff: −0.39). This indicates that while the LLM often excels in JSD, its advantage is not universal and can be pool-dependent, with RF sometimes performing better especially with purely demographic information.

Regarding Cramér’s V (Figure 5), which measures associative strength, the RF model again tends to show stronger performance or parity in many topic–pool combinations. Negative values, indicating RF superiority, are prominent for “Gun Regulation” across all pools, with the largest differences seen with D+M (Diff: −0.33), D (Diff: −0.22), and A+M (Diff: −0.21). For “Race Diversity”, the RF also consistently outperforms the LLM. The LLM demonstrates a competitive or superior Cramér’s V for “Drug Addiction” across most pools (e.g., M pool, Diff: +0.07) and for “Health Insurance Policy” with the M pool (Diff: +0.11). However, for many other topic–pool cells, the differences are small or slightly favor the RF model.

This head-to-head comparison, where both models process the exact same variables from each pool, suggests that the RF model often exhibits superior or comparable performance in terms of F1-score and Cramér’s V. The Gemma3 12B LLM, however, frequently demonstrates an advantage in capturing the distributional nuances of survey responses, as measured by JSD, although this strength can also be influenced by the specific variable pool provided.

## 5. Conclusions

Our study investigated the capabilities of LLMs, specifically Gemma3 12B, to act as synthetic survey respondents, utilizing the 2020 ANES dataset. Our findings demonstrate that LLMs, when appropriately conditioned, serve as a potent tool for simulating public opinion, significantly outperforming naive baseline models even with minimal input.

While a direct quantitative comparison of our performance metrics with those in the literature is challenging due to differing experimental setups, we can contextualize our findings against key benchmarks. For instance, Argyle et al. (2022), in their seminal work on “silicon sampling,” used eleven ANES 2016 variables to predict a twelfth. They measured the correspondence between the associative patterns in human and GPT-3 data using Cramér’s V, finding a “stunning correspondence” with a mean difference of only −0.026. Similarly, other recent work [20] has reported high LLM accuracy in predicting the U.S. presidential election. However, we argue that the predictive power in such tasks can be heavily driven by the inclusion of exceptionally strong proxy variables. For instance, a respondent’s political ideology or state of residence are already such powerful predictors of vote choice that they can mask the model’s true simulation capability. We build on this foundation by tasking the LLM with a more complex challenge: simulating opinions on politically charged topics like gun regulation, refugee policy, and racial diversity.

A key contribution of our work is the detailed comparative analysis against traditional Random Forest classifiers and the nuanced exploration of different categories of backstory information—Demographic, Attitudinal and Political Orientation, and Moral and Social Values. We consistently found that while LLMs achieve individual-level prediction accuracy (F1-score) comparable to Random Forests, they notably excel in capturing the overall distributional patterns of opinions (as measured by Jensen–Shannon Distance) across a majority of socio-political topics. This superior performance in reflecting collective sentiment is particularly evident when LLMs are provided with richer combinations of attitudinal and moral variables, often surpassing the utility of demographic data alone. This highlights why previous studies focusing primarily on demographic conditioning may have observed limited steerability.

The research also explored the LLM’s inherent ability to discern relevant variables, showing that even when tasked with selecting a subset of features (Experiment 3), performance remained robust and, in some cases, JSD improved. This suggests a potential for more efficient prompting strategies, though with the caveat that crucial, topic-specific variables might occasionally be overlooked if not explicitly provided.

In direct head-to-head comparisons with Random Forests using identical input variable pools (Experiment 4), the pattern held: Random Forests often showed a slight edge or parity in F1-score and Cramér’s V, while the LLM maintained its advantage in distributional similarity (JSD). This underscores distinct strengths, with RFs suited for raw predictive power on individual responses and LLMs better at simulating the collective opinion landscape.

We hypothesize that these distinct strengths stem directly from the models’ training paradigms. The Random Forest is a discriminative model trained exclusively to find the optimal predictive pathways within the ANES sample, making it highly proficient at individual classification (F1-score, Cramér’s V). Conversely, the LLM leverages its vast pre-training on web-scale text, which has implicitly taught it the statistical distributions of beliefs and attitudes at a population level. This allows it to generate responses that better reflect the overall shape of public opinion, often leading to superior distributional accuracy (JSD).

To ensure these observations are not an artifact of a single model, we validated our findings through a full replication using Qwen2.5 14B (see Appendix B for summary tables). The results from Qwen2.5 consistently reinforce our central conclusion: while the supervised Random Forest model often holds a slight advantage in individual prediction accuracy (F1-score), an LLM excels at capturing the distributional characteristics of public opinion (JSD). This core trade-off was evident across both models. We did, however, observe minor differences in their capabilities. Gemma3 12B appeared to be a more competitive individual-level predictor, occasionally matching or exceeding the RF’s F1-score, whereas Qwen2.5’s performance lagged further behind the RF on this metric. Ultimately, the remarkable consistency in the type of strengths and weaknesses exhibited by two distinct LLMs strongly suggests that this is a fundamental distinction between generative simulation and supervised classification.

In conclusion, our research substantiates the growing potential of LLMs as a valuable and scalable instrument for social science research. They offer a promising, cost-effective method to generate synthetic survey data, explore “silicon sampling”, and complement traditional survey methodologies, especially for understanding nuanced collective opinion patterns. While traditional machine learning models like Random Forests may retain advantages in specific individual prediction contexts, the demonstrated strength of LLMs in distributional modeling positions them as an important new tool for advancing our understanding of public opinion dynamics.

## 6. Limitations and Future Research

While this study provides strong evidence for the utility of LLMs in public opinion simulation, it is important to acknowledge its limitations. First, our analysis is centered on a single, small-sized open-source model, Gemma3 12B. Performance and behavioral patterns may vary with different model architectures, sizes (e.g., larger proprietary models like GPT-4), or quantization methods. Future work should extend this comparative framework to a broader range of models to assess the generalizability of our findings.

Second, the findings are rooted in the 2020 American National Election Studies (ANES) dataset. The observed relationships between demographic, attitudinal, and moral variables are specific to the U.S. socio-political context of that time. The framework’s effectiveness in simulating public opinion in other cultures, political systems, or time periods requires explicit validation.

Third, while we described our prompt templates and temperature settings in the methods section, we acknowledge that prompt design choices can meaningfully affect model behavior. Subtle changes in wording, formatting, or ordering of variables can lead to shifts in model outputs—particularly in settings involving moral or ideological framing. While prior work has found such simulations to be relatively robust across a wide range of temperature values, prompt sensitivity remains an open methodological challenge in computational social science. Future work should explore more systematic sensitivity analyses and the development of prompt-invariant methods to ensure the robustness and reproducibility of simulation results.

Finally, this work is confined to simulating responses for structured, multiple-choice survey questions. While our distributional metrics (JSD) show strong aggregate performance, there remains a risk that LLMs can generate “caricatures” that over-represent common statistical correlations rather than capturing the full spectrum of nuanced or atypical human beliefs. The ability to generate authentic, open-ended textual responses remains a key area for future exploration. These limitations highlight important directions for continued research, including cross-cultural validation and the development of more sophisticated metrics for assessing the qualitative authenticity of synthetic personas.

## Figures and Tables

**Table 1:** Demographic backstory variables.

Variable	Choices
Race	1. White 2. Black 3. Hispanic 4. Asian 5. Native American 6. Mixed
Age	*[free form value]*
Gender	1. Male 2. Female
Income (all family)	1 Under USD 9,999 2. USD 10,000–14,999 3. USD 15,000–19,999 … 15. USD 80,000–89,999 16. USD 90,000–99,999 17. USD 100,000–109,999 … 21. USD 175,000–249,999 22. USD 250,000 or more
Education	1. Less than high school credential 2. High school credential 3. Some post-high school, no bachelor’s degree 4. Bachelor’s degree 5. Graduate degree
Occupation	1. For-profit company or organization 2. Non-profit organization 3. Local government 4. State government 5. Military 6. Federal government, as a civilian employee 7. Owner of non-incorporated business 8. Owner of incorporated business 9. for-profit family business
City or rural	1. City person 2. Suburb person 3. Small-town person 4. Country person 5. Neither a city nor rural person
Children	0. No children 1. One child 2. Two children 3. Three children 4. Four or more children
Has health insurance	1. Yes 2. No

**Table 2:** Attitudinal backstory variables.

Variable	Choices
Ideology	1. Extremely liberal 2. Liberal 3. Slightly liberal 4. Moderate 5. Slightly conservative 6. Conservative 7. Extremely conservative
Party	1. Strong Democrat 2. Not very strong Democrat 3. Independent who leans Democratic 4. Independent 5. Independent who leans Republican 6. Not very strong Republican 7. Strong Republican
Interested in politics	1. Very interested 2. Somewhat interested 3. Not very interested 4. Not at all interested
Trust Media	1. No 2. A little 3. A moderate amount 4. A lot 5. A great deal
Vaccines & Autism	1. Most scientific evidence shows childhood vaccines cause autism 2. Most scientific evidence shows childhood vaccines do not cause autism
Science Experts Necessity	1. Do not need 2. Need a little 3. Need a moderate amount 4. Need a lot 5. Need a great deal
Religion importance	1. Extremely important 2. Very important 3. Moderately important 4. Little importance 5. Not important at all

**Table 3:** Moral and social values backstory variables.

Variable	Choices
Preferred Child Trait	1. Obedience 2. Self-reliance 3. Both 4. Neither
Death Penalty	1. Favor 2. Oppose
Birthright Citizenship End	1. Favor a great deal 2. Favor a moderate amount 3. Favor a little 4. Neither favor nor oppose 5. Oppose a little 6. Oppose a moderate amount 7. Oppose a great deal
Children Deportation	1. Favor a great deal 2. Favor a moderate amount 3. Favor a little 4. Oppose a little. 5. Oppose a moderate amount 6. Oppose a great deal
Discrimination Women	1. A great deal 2. A lot 3. A moderate amount 4. A little 5. None
Discrimination Black	1. A great deal 2. A lot 3. A moderate amount 4. A little 5. None
Discrimination Gays	1. A great deal 2. A lot 3. A moderate amount 4. A little 5. None
Discrimination Muslims	1. A great deal 2. A lot 3. A moderate amount 4. A little 5. None
Historic Racism Impact	1. Agree strongly 2. Agree somewhat 3. Neither agree nor disagree 4. Disagree somewhat 5. Disagree strongly

**Table 4:** Survey topics.

Variable	Question	Choices
Race diversity	Does the increasing number of people of many different races and ethnic groups in the United States make this country a better place to live, a worse place to live, or does it make no difference?	1. Better 2. Worse 3. Makes no difference
Gender role	Do you think it is better, worse, or makes no difference for the family as a whole if the man works outside the home and the woman takes care of the home and family?	1. Better 2. Worse 3. Makes no difference
Current Economy*	What do you think about the state of the economy these days in the United States?	1. Good 2. Neither good nor bad 3. Bad
Drug addiction	Do you think the federal government should be doing more about the opioid drug addiction issue, should be doing less, or is it currently doing the right amount?	1. Should be doing more 2. Should be doing less 3. Is doing the right amount
Climate change*	How much, if at all, do you think climate change is currently affecting severe weather events or temperature patterns in the United States?	1. Not at all 2. A little 3. A lot
Gay marriage	Which comes closest to your view regarding gay and lesbian couples?	1. They should be allowed to legally marry 2. They should be allowed to form civil unions but not legally marry 3. There should be no legal recognition of gay or lesbian couples relationship
Refugee allowing	Do you favor, oppose, or neither favor nor oppose allowing refugees who are fleeing war, persecution, or natural disasters in other countries to come to live in the U.S.?	1. Favor 2. Oppose 3. Neither favor nor oppose
Health insurance Policy	Do you favor an increase, decrease, or no change in government spending to help people pay for health insurance when people cannot pay for it all themselves?	1. Increase 2. Decrease 3. No change
Gun regulation	Do you think the federal government should make it more difficult for people to buy a gun than it is now, make it easier for people to buy a gun, or keep these rules about the same as they are now?	1. More difficult 2. Easier 3. Keep these rules about the same
Income inequality	Do you favor, oppose, or neither favor nor oppose the government trying to reduce the difference in incomes between the richest and poorest households?	1. Favor 2. Oppose 3. Neither favor nor oppose

**Table 5:** Performance comparison between Gemma3 12B and baseline models for Experiment 1. Reported metrics are averaged across folds. Lower Jensen–Shannon distance (JSD) indicates better distributional alignment; higher Cramér’s V and F1-score indicate stronger predictive and associative performance. The bold highlights best-performing value for each metric and topic.

Topic	Model	F1-Score	JSD	Cramér’s V
*Climate Change*	Gemma3 12B	**0.59**	**0.21**	**0.41**
	Random	0.36	0.29	0.04
	Constant	0.41	0.51	-
*Current Economy*	Gemma3 12B	**0.52**	**0.08**	**0.29**
	Random	0.35	0.11	0.04
	Constant	0.29	0.59	-
*Drug Addiction*	Gemma3 12B	0.56	**0.20**	**0.10**
	Random	0.40	0.37	0.04
	Constant	**0.58**	0.41	-
*Gay Marriage*	Gemma3 12B	**0.68**	**0.10**	**0.30**
	Random	0.38	0.33	0.03
	Constant	0.59	0.40	-
*Gender Role*	Gemma3 12B	**0.61**	**0.12**	**0.20**
	Random	0.38	0.34	0.04
	Constant	0.52	0.44	-
*Gun Regulation*	Gemma3 12B	**0.59**	**0.25**	**0.29**
	Random	0.38	0.30	0.03
	Constant	0.38	0.53	-
*Health Insurance Policy*	Gemma3 12B	**0.59**	**0.23**	**0.29**
	Random	0.36	0.24	0.03
	Constant	0.42	0.51	-
*Income Inequality*	Gemma3 12B	**0.61**	**0.06**	**0.40**
	Random	0.35	0.12	0.03
	Constant	0.32	0.57	-
*Race Diversity*	Gemma3 12B	**0.60**	**0.19**	**0.21**
	Random	0.37	0.32	0.03
	Constant	0.42	0.51	-
*Refugee Allowing*	Gemma3 12B	**0.47**	0.37	**0.25**
	Random	0.36	**0.22**	0.03
	Constant	0.40	0.52	-

**Table 6:** Gemma3 12B’s best performance across variable pools in Experiment 2. For each survey question and metric, the table shows the variable pool that yielded the peak performance, along with the corresponding best F1-score, JSD, and Cramer’s V.

Topic	F1-Score	JS	Cramér’s V	F1-Score Pool	JS Pool	Cramér’s V Pool
Climate Change	0.70	0.15	0.44	A+M	A+M	A+M
Current Economy	0.53	0.08	0.35	A	A	A+M
Drug Addiction	0.62	0.12	0.12	M	A	M
Gay Marriage	0.66	0.19	0.31	A	A	A
Gender Role	0.63	0.05	0.20	D+A+M	A	D+A
Gun Regulation	0.62	0.20	0.35	A	A	D+A
Health Insurance Policy	0.62	0.06	0.39	D+A+M	D+A	M
Income Inequality	0.61	0.10	0.42	A+M	A	A+M
Race Diversity	0.60	0.08	0.26	A	A	D+A
Refugee Allowing	0.57	0.11	0.30	A	A	D+A+M

**Table 7:** RF’s best performance across variable pools in Experiment 2. For each survey question and metric, the table shows the variable pool that yielded the peak performance, along with the corresponding best F1-score, JSD, and Cramer’s V.

Topic	F1-Score	JS	Cramér’s V	F1-Score Pool	JS Pool	Cramér’s V Pool
Climate Change	0.72	0.12	0.46	A+M	M	A+M
Current Economy	0.56	0.15	0.37	A+M	M	A+M
Drug Addiction	0.59	0.30	0.06	A+M	M	D+A+M
Gay Marriage	0.69	0.15	0.30	D+A	A	A+M
Gender Role	0.62	0.23	0.22	A+M	A	D+A+M
Gun Regulation	0.70	0.16	0.53	D+A+M	M	D+A+M
Health Insurance Policy	0.62	0.18	0.33	A+M	M	A+M
Income Inequality	0.61	0.13	0.41	A+M	A	A+M
Race Diversity	0.65	0.15	0.43	D+A+M	M	D+A+M
Refugee Allowing	0.62	0.11	0.35	A+M	M	A+M

**Table 8:** Performance gains across variable pools for Experiment 2 when results from Table 6 are compared to the LLM baseline in Experiment 1 (Table 5). Gains are reported as the difference [Experiment 2 − Experiment 1] for F1-score and Cramer’s V and [Experiment 1 − Experiment 2] for JSD, so that positive values/blue shades consistently favor Experiment 2. The colors clearly show the performance pattern between the models. The * marks the statistical significance of the metric value.

Topic	Gain F1-Score	Gain JS	Gain Cramér’s V
Climate Change	0.11 *	0.07 *	0.03 *
Current Economy	0.02 *	0.00	0.06 *
Drug Addiction	0.06 *	0.09 *	0.02
Gay Marriage	−0.02 *	−0.09 *	0.02
Gender Role	0.02 *	0.07 *	−0.00
Gun Regulation	0.04 *	0.05 *	0.05 *
Health Insurance Policy	0.03 *	0.17 *	0.10 *
Income Inequality	0.00	−0.04 *	0.02 *
Race Diversity	0.00	0.11 *	0.05 *
Refugee Allowing	0.09 *	0.26 *	0.05 *

**Table 9:** Performance differences across variable pools for Experiment 2 when results from Table 6 are compared to the peak RF performance from Table 7. Differences are calculated as [Peak LLM score − Peak RF score] for F1-score and Cramer’s V, and [Peak RF JSD − Peak LLM JSD] for JSD, so that positive values/blue shades consistently favor the LLM. The colors clearly show the performance pattern between the models. The * marks the statistical significance of the metric value.

Topic	Diff F1-Score	Diff JS	Diff Cramér’s V
Climate Change	−0.02 *	−0.02 *	−0.02
Current Economy	−0.03 *	0.07 *	−0.02 *
Drug Addiction	0.02 *	0.18 *	0.06 *
Gay Marriage	−0.03 *	−0.04 *	0.02
Gender Role	0.01 *	0.18 *	−0.03
Gun Regulation	−0.08 *	−0.04 *	−0.19 *
Health Insurance Policy	0.00	0.12 *	0.06 *
Income Inequality	0.00	0.03 *	0.01
Race Diversity	−0.05 *	0.08 *	−0.17 *
Refugee Allowing	−0.06*	−0.00	−0.04 *

**Table 10:** Gemma3 12B’s best performance across variable pools in Experiment 3. For each survey question and metric, the table shows the variable pool that yielded the peak performance, along with the corresponding best F1-score, JSD, and Cramer’s V. Here we instruct the LLM to perform Feature Selection.

Topic	F1-Score	JS	Cramér’s V	F1-Score Pool	JS Pool	Cramér’s V Pool
Climate Change	0.69	0.09	0.42	D+A	D+A	D+A+M
Current Economy	0.55	0.06	0.36	A+M	A	A
Drug Addiction	0.63	0.07	0.15	D+M	D+A	D+M
Gay Marriage	0.66	0.16	0.31	D+A+M	A	D+A
Gender Role	0.60	0.08	0.23	D+A+M	D+A+M	D+A+M
Gun Regulation	0.63	0.18	0.36	D+A	A+M	A
Health Insurance Policy	0.58	0.17	0.34	D+M	A+M	D+M
Income Inequality	0.62	0.02	0.41	A	A	A
Race Diversity	0.60	0.03	0.25	D+A	D+A	D+A
Refugee Allowing	0.56	0.14	0.29	D+A	D	D+A

**Table 11:** Comparison of peak LLM performance from Experiment 3 and peak RF performance from Experiment 2. Here we instruct the LLM to perform Feature Selection. The “Diff” columns show the performance difference relative to the best LLM result (from Table 6) for the corresponding topic and metric. Differences are calculated as [Peak LLM score–Peak RF score] for F1-score and Cramer’s V, and [Peak RF JSD–Peak LLM JSD] for JSD, so that positive values/blue shades consistently favor the LLM. The colors clearly show the performance pattern between the models. The * marks the statistical significance of the metric value.

Topic	Diff F1-Score	Diff JS	Diff Cramér’s V
Climate Change	−0.03 *	0.04 *	−0.04 *
Current Economy	−0.01	0.08 *	−0.01
Drug Addiction	0.04 *	0.23 *	0.09 *
Gay Marriage	−0.03 *	−0.01	0.02
Gender Role	−0.01	0.15 *	0.00
Gun Regulation	−0.07 *	−0.02	−0.18 *
Health Insurance Policy	−0.04 *	0.02	0.01
Income Inequality	0.01	0.12 *	0.00
Race Diversity	−0.05 *	0.12 *	−0.18 *
Refugee Allowing	−0.07 *	−0.04 *	−0.05 *

## Data Availability

The code and data used in this study are publicly available at https://github.com/fernandotenorio/ANES_2020_survey_simulation (accessed on 4 August 2025).

## Appendix A. Prompts

### Appendix A.1. Baseline Feature Selection

*You are a reasoning assistant trained to understand how personal characteristics shape beliefs and opinions. Your task is to analyze a set of variables describing individuals in the U.S. and select the variables most predictive of a person’s response to a survey question on the topic of {TOPIC}. Below are the variables grouped by theme and their descriptions.*

**Demographic**
*race*: Self-identified race
*age*: Age
*gender*: Male or female
*income*: Annual family income
*education*: Education level
*occupation*: Employment type
*city_rural*: Identifies as urban or rural
*children*: Number of children
*health_insurance*: Has health insurance or not

**Attitudes & Political Orientation**
*ideology*: Political ideology (liberal, conservative, etc.)
*party*: Political party affiliation
*interested_politics*: Interested in politics or not
*trust_media*: Trust in mainstream media
*science*: Belief that people need help from experts to understand science
*vaccines_autism*: Belief that vaccines cause autism
*religion_importance*: How important religion is in the respondent’s life

**Moral Compass & Social Values**
*child_trait*: Most important child trait (e.g., obedience vs. self-reliance)
*death_penalty*: Support or opposition to the death penalty
*birth_citizenship*: View on banning birthright citizenship for children of undocumented immigrants
*children_sent_back*: View on deporting children of undocumented immigrants
*discrimination_woman*: Perceived discrimination against women
*black_discrimination*: Perceived discrimination against Black people
*black_hist*: Belief that past racism still affects Black Americans today
*gays_discrimination*: Perceived discrimination against gay people
*muslims_discrimination*: Perceived discrimination against Muslims

**Survey Question**
We want to predict the respondent’s answer to the following question:

**Question:**
{QUESTION}

**Answer choices:**
{ANSWER CHOICES}

**Instructions**
Based on your understanding of human behavior, select **exactly one variable from each of the three groups** above that you believe is the most predictive of how someone would respond to the question above. Respond only in **this JSON format**:
{
"selected_variables": ["variable_1", "variable_2", "variable_3"]
}

### Appendix A.2. Variables Pool Feature Selection

*You are a reasoning assistant trained to understand how personal characteristics shape beliefs and opinions. Your task is to analyze a set of variables describing individuals in the U.S. and select the variables most predictive of a person’s response to a survey question on the topic of {TOPIC}. Below are the variables grouped by theme and their descriptions.*

**Demographic**
*race*: Self-identified race
*age*: Age
*gender*: Male or female
*income*: Annual family income
*education*: Education level
*occupation*: Employment type
*city_rural*: Identifies as urban or rural
*children*: Number of children
*health_insurance*: Has health insurance or not

**Attitudes & Political Orientation**
*ideology*: Political ideology (liberal, conservative, etc.)
*party*: Political party affiliation
*interested_politics*: Interested in politics or not
*trust_media*: Trust in mainstream media
*science*: Belief that people need help from experts to understand science
*vaccines_autism*: Belief that vaccines cause autism
*religion_importance*: How important religion is in the respondent’s life

**Moral Compass & Social Values**
*child_trait*: Most important child trait (e.g., obedience vs. self-reliance)
*death_penalty*: Support or opposition to the death penalty
*birth_citizenship*: View on banning birthright citizenship for children of undocumented immigrants
*children_sent_back*: View on deporting children of undocumented immigrants
*discrimination_woman*: Perceived discrimination against women
*black_discrimination*: Perceived discrimination against Black people
*black_hist*: Belief that past racism still affects Black Americans today
*gays_discrimination*: Perceived discrimination against gay people
*muslims_discrimination*: Perceived discrimination against Muslims

**Survey Question**
We want to predict the respondent’s answer to the following question:
**Question:**
{QUESTION}

**Answer choices:**
{ANSWER CHOICES}

**Instructions**
Based on your understanding of human behavior, select **only the variables that are most predictive** of how someone would respond to the question above. Favor **clarity and precision** over length. Respond only in **this JSON format**:
{
"selected_variables": ["variable_1", "variable_2", "…"]
}

### Appendix A.3. Baseline Feature Selection: Gun Regulation Example

*You are a reasoning assistant trained to understand how personal characteristics shape beliefs and opinions. Your task is to analyze a set of variables describing individuals in the U.S. and select the variables most predictive of a person’s response to a survey question on the topic of “Gun regulation”. Below are the variables grouped by theme and their descriptions.*

**Demographic**
*race*: Self-identified race
*age*: Age
*gender*: Male or female
*income*: Annual family income
*education*: Education level
*occupation*: Employment type
*city_rural*: Identifies as urban or rural
*children*: Number of children
*health_insurance*: Has health insurance or not

**Attitudes & Political Orientation**
*ideology*: Political ideology (liberal, conservative, etc.)
*party*: Political party affiliation
*interested_politics*: Interested in politics or not
*trust_media*: Trust in mainstream media
*science*: Belief that people need help from experts to understand science
*vaccines_autism*: Belief that vaccines cause autism
*religion_importance*: How important religion is in the respondent’s life

**Moral Compass & Social Values**
*child_trait*: Most important child trait (e.g., obedience vs. self-reliance)
*death_penalty*: Support or opposition to the death penalty
*birth_citizenship*: View on banning birthright citizenship for children of undocumented immigrants
*children_sent_back*: View on deporting children of undocumented immigrants
*discrimination_woman*: Perceived discrimination against women
*black_discrimination*: Perceived discrimination against Black people
*black_hist*: Belief that past racism still affects Black Americans today
*gays_discrimination*: Perceived discrimination against gay people
*muslims_discrimination*: Perceived discrimination against Muslims

**Survey Question**
We want to predict the respondent’s answer to the following question:

**Question:**
Do you think the federal government should make it more difficult for people to buy a gun than it is now, make it easier for people to buy a gun, or keep these rules about the same as they are now?

**Answer choices:**
(1) More difficult (2) Easier (3) Keep these rules about the same
**Instructions**
Based on your understanding of human behavior, select **exactly one variable from each of the three groups** above that you believe is the most predictive of how someone would respond to the question above. Respond only in **this JSON format**:
{
"selected_variables": ["variable_1", "variable_2", "variable_3"]
}

### Appendix A.4. Variables Pool Feature Selection: Gun Regulation Example

*You are a reasoning assistant trained to understand how personal characteristics shape beliefs and opinions. Your task is to analyze a set of variables describing individuals in the U.S. and select the variables most predictive of a person’s response to a survey question on the topic of “Gun regulation”. Below are the variables grouped by theme and their descriptions.*

**Demographic**
*race*: Self-identified race
*age*: Age
*gender*: Male or female
*income*: Annual family income
*education*: Education level
*occupation*: Employment type
*city_rural*: Identifies as urban or rural
*children*: Number of children
*health_insurance*: Has health insurance or not

**Attitudes & Political Orientation**
*ideology*: Political ideology (liberal, conservative, etc.)
*party*: Political party affiliation
*interested_politics*: Interested in politics or not
*trust_media*: Trust in mainstream media
*science*: Belief that people need help from experts to understand science
*vaccines_autism*: Belief that vaccines cause autism
*religion_importance*: How important religion is in the respondent’s life

**Moral Compass & Social Values**
*child_trait*: Most important child trait (e.g., obedience vs. self-reliance)
*death_penalty*: Support or opposition to the death penalty
*birth_citizenship*: View on banning birthright citizenship for children of undocumented immigrants
*children_sent_back*: View on deporting children of undocumented immigrants
*discrimination_woman*: Perceived discrimination against women
*black_discrimination*: Perceived discrimination against Black people
*black_hist*: Belief that past racism still affects Black Americans today
*gays_discrimination*: Perceived discrimination against gay people
*muslims_discrimination*: Perceived discrimination against Muslims

**Survey Question**
We want to predict the respondent’s answer to the following question:

**Question:**
Do you think the federal government should make it more difficult for people to buy a gun than it is now, make it easier for people to buy a gun, or keep these rules about the same as they are now?

**Answer choices:**
(1) More difficult (2) Easier (3) Keep these rules about the same
**Instructions**
Based on your understanding of human behavior, select **only the variables that are most predictive** of how someone would respond to the question above. Favor **clarity and precision** over length. Respond only in **this JSON format**:
{
"selected_variables": ["variable_1", "variable_2", "…"]
}

### Appendix A.5. Survey

*Roleplay the person below. The date is 3 November 2020. When questioned, answer just with the option number and nothing more.*

*{SELECTED BACKSTORY VARIABLE 1.}*
*{SELECTED BACKSTORY VARIABLE 2.}*
*{…}*
*{SELECTED BACKSTORY VARIABLE N.}*

**Question:**
{QUESTION}
**Answer choices:**

{ANSWER CHOICES}

*My answer is*

### Appendix A.6. Survey: Gun Regulation Example with All Backstory Variables

*Roleplay the person below. The date is 3 November 2020. When questioned, answer just with the option number and nothing more.*

*Racially, you are White.*
*Ideologically, you are slightly conservative.*
*Politically, you are not very strong Republican.*
*Religion is moderately important in my life.*
*You are 42 years old.*
*You are a man.*
*You are not at all interested in politics.*
*Your total family income is $175,000–249,999.*
*At the moment you do have health insurance.*
*You have a Bachelor’s degree.*
*Your employment is best described as working for a for-profit company or organization.*
*You consider yourself a small-town person.*
*You have two children.*
*You believe that people need a lot of help from experts to understand science.*
*You believe that most scientific evidence shows childhood vaccines do not cause autism.*
*You have little trust in the media when it comes to reporting the news accurately and fairly.*
*You believe that when it comes to obedience vs. self-reliance, obedience is a more important trait for a child to have.*
*You believe there is a moderate amount of discrimination against women in the US today.*
*You favor a moderate amount that children of unauthorized immigrants do not automatically get citizenship if they are born in the US.*
*You oppose a little that children of unauthorized immigrants who were brought to the US illegally and have lived here for at least 10 years should be deported.*
*You disagree somewhat that generations of slavery and discrimination have created conditions that make it difficult for blacks to work their way out of the lower class.*
*You believe there is a moderate amount of discrimination against blacks in the US today.*
*You believe there is a moderate amount of discrimination against gays and lesbians in the US today.*
*You believe there is a lot of discrimination against muslims in the US today.*
*You favor the death penalty.*

**Question:**
Do you think the federal government should make it more difficult for people to buy a gun than it is now, make it easier for people to buy a gun, or keep these rules about the same as they are now?

**Answer choices:**
(1) More difficult (2) Easier (3) Keep these rules about the same

*My answer is*

## Appendix B. 95% Bootstrap Confidence Intervals for Performance Metrics

All estimates are based on B=1000 bootstrap iterations.

### Appendix B.1. Experiment 1: Gemma3 12B Baseline

**Table A1:** 95% CIs for Gemma3 12B baseline performance across topics in Experiment 1. Point estimates are provided in Table 5.

Topic	F1-Score	JS	Cramér’s V
Climate Change	[0.574, 0.604]	[0.201, 0.228]	[0.394, 0.435]
Current Economy	[0.5, 0.531]	[0.071, 0.1]	[0.266, 0.31]
Drug Addiction	[0.541, 0.573]	[0.187, 0.221]	[0.087, 0.133]
Gay Marriage	[0.665, 0.698]	[0.083, 0.119]	[0.278, 0.323]
Gender Role	[0.592, 0.623]	[0.1, 0.133]	[0.182, 0.227]
Gun Regulation	[0.569, 0.601]	[0.237, 0.269]	[0.276, 0.312]
Health Insurance Policy	[0.574, 0.608]	[0.218, 0.246]	[0.268, 0.313]
Income Inequality	[0.592, 0.622]	[0.049, 0.078]	[0.377, 0.419]
Race Diversity	[0.584, 0.617]	[0.175, 0.205]	[0.196, 0.254]
Refugee Allowing	[0.456, 0.491]	[0.35, 0.383]	[0.232, 0.275]

### Appendix B.2. Experiment 1: Gemma3 12B Baseline Gains Against Random Model

**Table A2:** 95% CIs for Gemma3 12B baseline performance gains over the Random model in Experiment 1. Point estimates are provided in Table 5. The “*” marks the statistica significance of the metric value.

Topic	Gain F1-Score	Gain JS	Gain Cramér’s V
Climate change	[0.201, 0.246] *	[0.055, 0.094] *	[0.336, 0.39] *
Current economy	[0.144, 0.188] *	[0.001, 0.055] *	[0.203, 0.263] *
Drug addiction	[0.134, 0.178] *	[0.153, 0.192] *	[0.028, 0.088] *
Gay marriage	[0.277, 0.319] *	[0.204, 0.255] *	[0.224, 0.284] *
Gender role	[0.201, 0.246] *	[0.201, 0.251] *	[0.117, 0.178] *
Gun regulation	[0.184, 0.23] *	[0.027, 0.077] *	[0.22, 0.271] *
Health insurance policy	[0.205, 0.248] *	[−0.016, 0.038]	[0.214, 0.271] *
Income inequality	[0.234, 0.276] *	[0.037, 0.083] *	[0.324, 0.377] *
Race diversity	[0.213, 0.254] *	[0.103, 0.159] *	[0.141, 0.212] *
Refugee allowing	[0.096, 0.14] *	[−0.169, −0.122] *	[0.181, 0.236] *

### Appendix B.3. Experiment 1: Gemma3 12B Baseline Gains Against Constant Model

**Table A3:** 95% CIs for Gemma3 12B baseline performance gains over the Constant model in Experiment 1. Point estimates are provided in Table 5. The “*” marks the statistica significance of the metric value.

Topic	Gain F1-Score	Gain JS	Gain Cramér’s V
Climate change	[0.15, 0.198] *	[0.272, 0.313] *	-
Current economy	[0.206, 0.247] *	[0.488, 0.514] *	-
Drug addiction	[−0.036, −0.004] *	[0.188, 0.232] *	-
Gay marriage	[0.076, 0.105] *	[0.284, 0.322] *	-
Gender role	[0.066, 0.108] *	[0.308, 0.349] *	-
Gun regulation	[0.181, 0.23] *	[0.255, 0.299] *	-
Health insurance policy	[0.157, 0.191] *	[0.26, 0.289] *	-
Income inequality	[0.272, 0.308] *	[0.486, 0.523] *	-
Race diversity	[0.163, 0.199] *	[0.303, 0.329] *	-
Refugee allowing	[0.06, 0.086] *	[0.133, 0.165] *	-

### Appendix B.4. Experiment 2: Gemma3 12B Best Performance

**Table A4:** 95% CIs for Gemma3 12B best performance in Experiment 2. Point estimates are provided in Table 6.

Topic	F1-Score	JS	Cramér’s V
Climate Change	[0.681, 0.709]	[0.131, 0.161]	[0.42, 0.461]
Current Economy	[0.52, 0.55]	[0.065, 0.096]	[0.33, 0.369]
Drug Addiction	[0.599, 0.638]	[0.1, 0.135]	[0.093, 0.161]
Gay Marriage	[0.649, 0.68]	[0.17, 0.206]	[0.295, 0.339]
Gender Role	[0.616, 0.647]	[0.034, 0.068]	[0.176, 0.223]
Gun Regulation	[0.608, 0.639]	[0.188, 0.219]	[0.326, 0.408]
Health Insurance Policy	[0.605, 0.636]	[0.047, 0.082]	[0.361, 0.415]
Income Inequality	[0.595, 0.624]	[0.088, 0.119]	[0.398, 0.435]
Race Diversity	[0.588, 0.621]	[0.062, 0.093]	[0.243, 0.285]
Refugee Allowing	[0.549, 0.582]	[0.093, 0.129]	[0.285, 0.327]

### Appendix B.5. Experiment 2: Gemma3 12B Performance Gains Against Experiment 1 LLM Baseline

**Table A5:** 95% CIs for performance gains when comparing Gemma3 12B against the Experiment 1 LLM baseline. Point estimates are provided in Table 8 in the main text. An asterisk (*) indicates a 95% CI for the gain that does not include zero. The “*” marks the statistica significance of the metric value.

Topic	Gain F1-Score	Gain JS	Gain Cramér’s V
Climate Change	[0.092, 0.122] *	[0.051, 0.085] *	[0.006, 0.047] *
Current Economy	[0.001, 0.035] *	[−0.016, 0.025]	[0.04, 0.082] *
Drug Addiction	[0.046, 0.079] *	[0.059, 0.109] *	[−0.021, 0.054]
Gay Marriage	[−0.028, −0.005] *	[−0.104, −0.071] *	[−0.004, 0.033]
Gender Role	[0.01, 0.038] *	[0.037, 0.098] *	[−0.025, 0.018]
Gun Regulation	[0.026, 0.051] *	[0.038, 0.061] *	[0.038, 0.115] *
Health Insurance Policy	[0.015, 0.043] *	[0.139, 0.195] *	[0.068, 0.128] *
Income Inequality	[−0.01, 0.016]	[−0.053, −0.028] *	[0.004, 0.037] *
Race Diversity	[−0.01, 0.019]	[0.095, 0.13] *	[0.012, 0.069] *
Refugee Allowing	[0.076, 0.108] *	[0.237, 0.273] *	[0.032, 0.072] *

### Appendix B.6. Experiment 2: Gemma3 12B Performance Diff Against RF Model in Experiment 2

**Table A6:** 95% CIs for performance differences when comparing Gemma3 12B against the RF model in Experiment 2. Point estimates are provided in Table 9 in the main text. An asterisk (*) indicates a 95% CI for the difference that does not include zero. The “*” marks the statistica significance of the metric value.

Topic	Diff F1-Score	Diff JS	Diff Cramér’s V
Climate Change	[−0.038, −0.011] *	[−0.038, −0.002] *	[−0.041, 0.001]
Current Economy	[−0.043, −0.007] *	[0.04, 0.097] *	[−0.038, −0.006] *
Drug Addiction	[0.015, 0.036] *	[0.163, 0.196] *	[0.012, 0.104] *
Gay Marriage	[−0.04, −0.012] *	[−0.06, −0.017] *	[−0.01, 0.042]
Gender Role	[0.001, 0.029] *	[0.156, 0.203] *	[−0.056, 0.009]
Gun Regulation	[−0.091, −0.062] *	[−0.06, −0.027] *	[−0.212, −0.121] *
Health Insurance Policy	[−0.01, 0.017]	[0.093, 0.15] *	[0.025, 0.083] *
Income Inequality	[−0.014, 0.016]	[0.003, 0.061] *	[−0.008, 0.024]
Race Diversity	[−0.065, −0.035] *	[0.052, 0.101] *	[−0.192, −0.143] *
Refugee Allowing	[−0.077, −0.043] *	[−0.032, 0.022]	[−0.066, −0.019] *

### Appendix B.7. Experiment 3: Gemma3 12B Best Performance

**Table A7:** 95% CIs for Gemma3 12B best performance in Experiment 3. Point estimates are provided in Table 10.

Topic	F1-Score	JS	Cramér’s V
Climate Change	[0.679, 0.708]	[0.069, 0.103]	[0.404, 0.447]
Current Economy	[0.533, 0.565]	[0.051, 0.081]	[0.339, 0.38]
Drug Addiction	[0.617, 0.65]	[0.052, 0.087]	[0.122, 0.189]
Gay Marriage	[0.647, 0.681]	[0.142, 0.175]	[0.295, 0.338]
Gender Role	[0.588, 0.618]	[0.067, 0.101]	[0.208, 0.25]
Gun Regulation	[0.616, 0.647]	[0.164, 0.194]	[0.338, 0.439]
Health Insurance Policy	[0.564, 0.597]	[0.152, 0.187]	[0.317, 0.403]
Income Inequality	[0.6, 0.63]	[0.013, 0.037]	[0.391, 0.43]
Race Diversity	[0.587, 0.619]	[0.022, 0.054]	[0.228, 0.271]
Refugee Allowing	[0.54, 0.573]	[0.126, 0.16]	[0.277, 0.318]

### Appendix B.8. Experiment 3: Gemma3 12B Performance Diff Against RF Model in Experiment 2

**Table A8:** 95% CIs for performance differences when comparing Gemma3 12B against the RF model in Experiment 2. Point estimates are provided in Table 11 in the main text. An asterisk (*) indicates a 95% CI for the difference that does not include zero. The “*” marks the statistica significance of the metric value.

Topic	Diff F1-Score	Diff JS	Diff Cramér’s V
Climate Change	[−0.04, −0.014] *	[0.021, 0.059] *	[−0.058, −0.016] *
Current Economy	[−0.027, 0.006]	[0.052, 0.111] *	[−0.029, 0.004]
Drug Addiction	[0.026, 0.051] *	[0.202, 0.251] *	[0.039, 0.128] *
Gay Marriage	[−0.04, −0.014] *	[−0.032, 0.018]	[−0.01, 0.04]
Gender Role	[−0.03, 0.005]	[0.121, 0.178] *	[−0.03, 0.039]
Gun Regulation	[−0.083, −0.054] *	[−0.037, 0.001]	[−0.2, −0.095] *
Health Insurance Policy	[−0.049, −0.024] *	[−0.013, 0.047]	[−0.013, 0.063]
Income Inequality	[−0.006, 0.021]	[0.088, 0.13] *	[−0.015, 0.016]
Race Diversity	[−0.065, −0.036] *	[0.094, 0.135] *	[−0.205, −0.154] *
Refugee Allowing	[−0.087, −0.052] *	[−0.061, −0.01] *	[−0.078, −0.029] *

### Appendix B.9. Qwen2.5 14B Best Pools in Experiment 2

**Table A9:** Qwen2.5 14B’s best performance across variable pools in Experiment 2. For each survey question and metric, the table shows the variable pool that yielded the peak performance, along with the corresponding best F1-score, JSD, and Cramer’s V.

Topic	F1-Score	JS	Cramér’s V	F1-Score Pool	JS Pool	Cramér’s V Pool
Climate Change	0.52	0.10	0.41	D	D	A+M
Current Economy	0.49	0.23	0.25	A	A	A
Drug Addiction	0.64	0.12	0.14	M	A	M
Gay Marriage	0.65	0.21	0.30	A	A	A
Gender Role	0.57	0.13	0.22	D+A	A	D+A+M
Gun Regulation	0.65	0.22	0.46	A	A	A
Health Insurance Policy	0.57	0.14	0.36	A	A	D+A+M
Income Inequality	0.61	0.10	0.42	A+M	A+M	A+M
Race Diversity	0.63	0.02	0.30	A	A	A+M
Refugee Allowing	0.60	0.03	0.34	A+M	D	A+M

### Appendix B.10. Qwen2.5 14B Peak Performance in Experiment 2 vs. Random Forest

**Table A10:** Comparison of peak Qwen2.5 14B performance from Experiment 2 and peak RF performance from Experiment 2. The colors clearly show the performance pattern between the models. The * marks the statistical significance of the metric value.

Topic	Diff F1-Score	Diff JS	Diff Cramér’s V
Climate Change	−0.20 *	0.02 *	−0.05 *
Current Economy	−0.07 *	−0.08 *	−0.12 *
Drug Addiction	0.04 *	0.17 *	0.08 *
Gay Marriage	−0.04 *	−0.06 *	0.01
Gender Role	−0.04 *	0.10 *	−0.00
Gun Regulation	−0.05 *	−0.06 *	−0.07 *
Health Insurance Policy	−0.04 *	0.04 *	0.03 *
Income Inequality	−0.00	0.03 *	0.01
Race Diversity	−0.03 *	0.14 *	−0.13 *
Refugee Allowing	−0.02 *	0.08 *	−0.01

### Appendix B.11. Qwen2.5 14B Best Pools in Experiment 3

**Table A11:** Qwen2.5 14B’s best performance across variable pools in Experiment 2. For each survey question and metric, the table shows the variable pool that yielded the peak performance, along with the corresponding best F1-score, JSD, and Cramer’s V. Here we instruct the LLM to perform Feature Selection.

Topic	F1-Score	JS	Cramér’s V	F1-Score Pool	JS Pool	Cramér’s V Pool
Climate Change	0.62	0.14	0.42	A	D	A+M
Current Economy	0.48	0.27	0.29	A	A+M	A
Drug Addiction	0.61	0.14	0.12	A	A	D+M
Gay Marriage	0.67	0.14	0.32	D+A+M	A	A
Gender Role	0.58	0.09	0.22	D+A	D+A	D+A+M
Gun Regulation	0.67	0.20	0.49	A+M	A+M	A+M
Health Insurance Policy	0.58	0.13	0.34	D+M	A+M	A+M
Income Inequality	0.61	0.11	0.42	D+A+M	D+M	D+A+M
Race Diversity	0.61	0.07	0.25	D+M	A	D+A
Refugee Allowing	0.59	0.08	0.30	A+M	A+M	A+M

### Appendix B.12. Qwen2.5 14B Peak Performance in Experiment 3 vs. Random Forest

**Table A12:** Comparison of peak Qwen2.5 14B performance from Experiment 3 and peak RF performance from Experiment 2. The colors clearly show the performance pattern between the models. The * marks the statistical significance of the metric value.

Topic	Diff F1-Score	Diff JS	Diff Cramér’s V
Climate Change	−0.10 *	−0.01 *	−0.04 *
Current Economy	−0.08 *	−0.12 *	−0.08 *
Drug Addiction	0.02 *	0.15 *	0.06 *
Gay Marriage	−0.02 *	0.01	0.03 *
Gender Role	−0.04 *	0.14 *	−0.01
Gun Regulation	−0.03 *	−0.04 *	−0.05 *
Health Insurance Policy	−0.04 *	0.05 *	0.01
Income Inequality	0.00	0.03 *	0.01
Race Diversity	−0.05 *	0.08 *	−0.18 *
Refugee Allowing	−0.03 *	0.02 *	−0.04 *

## Appendix C. Computational Environment and Software

All Large Language Model interactions were conducted via a locally hosted Ollama server. This server was running on a machine equipped with an NVIDIA GeForce RTX 3060 GPU, featuring 12 GB of VRAM. The specific LLMs employed were the Gemma3 12B model, identified within Ollama as gemma3:12b-it-q4_K_M, and the Qwen2.5 14B model, identified as qwen2.5:14b-instruct-q6_K. These are both instruction-tuned models, utilized in their quantized form (q4_K_M and q6_K, respectively), and were downloaded directly from the Ollama model library.

The Python (v3.11.4) programming language was used for all the coding and Random Forest model training. The primary libraries and their versions were as follows:

- scikit-learn==1.5.2
- scipy==1.14.1
- ollama==0.4.8

All computations and software executions were performed on a machine running the Windows 10 operating system.

## Appendix D. Random Forest Classifier Parameters

The Random Forest models used for classification tasks throughout all experiments were instantiated from scikit-learn’s RandomForestClassifier class. To ensure consistency and comparability across experiments, the following hyperparameters were fixed:

- n_estimators = 100: The number of trees in the forest.
- max_depth = 10: The maximum depth of each tree.
- min_samples_split = 2: The minimum number of samples required to split an internal node.
- min_samples_leaf = 2: The minimum number of samples required to be at a leaf node.
- random_state: A specific integer value was used for the random_state parameter in each experimental run to ensure reproducibility of the results.

These parameters were chosen based on preliminary experimentation, to provide a reasonable balance between model complexity and generalization. During these initial tests, increasing the *number of estimators* beyond 100 yielded no measurable gains in performance. On the other hand, increasing *max_depth* led to noticeable increases in variance across validation folds, indicating potential overfitting. The goal of this setting was to define a stable, conservative baseline classifier for comparison with the LLMs, avoiding overfitting the Random Forest for each specific topic.

## Appendix E. Mathematical Definitions of Metrics

This appendix provides the mathematical definitions for the F1-score, Jensen–Shannon Distance and Cramér’s V.

For the F1-score, we utilized the *f1_score* function from the *scikit-learn* library, specifically choosing the “weighted” option for the “average” parameter to appropriately handle potential class imbalances.

Jensen–Shannon Distance values were computed using the *jensenshannon* function within the *scipy* library. To ensure the distance is normalized between 0 and 1, we set the “base” parameter to 2.

Finally, Cramér’s V was determined using the *association* function, also from *scipy*. This was configured with the “method” parameter set to “Cramer” and with bias “correction” enabled (“True”).

### Appendix E.1. F1-Score

The F1-score is the harmonic mean of precision and recall. It is commonly used to evaluate the performance of binary and multi-class classification models.

For a binary classification task, or for a specific class in a multi-class task, we define the following:

- True Positives (TPs): The number of positive instances correctly classified as positive.
- False Positives (FPs): The number of negative instances incorrectly classified as positive (Type I error).
- False Negatives (FNs): The number of positive instances incorrectly classified as negative (Type II error).
- True Negatives (TNs): The number of negative instances correctly classified as negative.

**Precision** is the ratio of correctly predicted positive observations to the total predicted positive observations:

(A1) $$\mathrm{Precision}=\frac{TP}{TP+FP}.$$

If TP+FP=0, precision is typically defined as 0 (as per scikit-learn’s “zero_division = ‘warn’” or “0” setting if “zero_division = 0”).

**Recall** (also known as Sensitivity or True Positive Rate) is the ratio of correctly predicted positive observations to all observations in the actual class:

(A2) $$\mathrm{Recall}=\frac{TP}{TP+FN}.$$

If TP+FN=0, recall is typically defined as 0.

The **F1-score** is then calculated as follows:

(A3) $$F_{1}=2\cdot \frac{\mathrm{Precision}\cdot \mathrm{Recall}}{\mathrm{Precision}+\mathrm{Recall}}=\frac{2\cdot TP}{2\cdot TP+FP+FN}.$$

If Precision + Recall = 0, the F1-score is 0.

The F1-score is a special case of the more general $F_{\beta }$**-score**, where β is a parameter that controls the relative importance of recall over precision:

(A4) $$F_{\beta }=\left(1+\beta^{2}\right)\cdot \frac{\mathrm{Precision}\cdot \mathrm{Recall}}{(\beta^{2}\cdot \mathrm{Precision})+\mathrm{Recall}}.$$

The F1-score sets β=1, meaning precision and recall are equally weighted.

**Multi-class and Multi-label Settings (Scikit-learn approach):** When dealing with multi-class or multi-label classification, the F1-score (and precision/recall) for each class can be calculated, and then these per-class scores are combined using an averaging method. In our work we use the weighted average method, explained below.

- **Weighted average:** Calculates metrics for each class and finds their average, weighted by the number of true instances for each class (support). This accounts for class imbalance.
  (A5) $$F1_{\mathrm{weighted}}=\sum_{c=1}^{N_{c}}w_{c}\cdot F1_{c}.$$
  where $w_{c}=\frac{{\mathrm{support}}_{c}}{\sum_{k=1}^{N_{c}}{\mathrm{support}}_{k}}$ is the proportion of true instances belonging to class *c*.

**Properties:**

- F1-score ranges from 0 to 1.
- F1=1 indicates perfect precision and recall.
- F1=0 if either precision or recall (or both) is 0.
- It is useful when there is an uneven class distribution as it considers both false positives and false negatives.

### Appendix E.2. Jensen–Shannon Distance (JSD)

Throughout the text we use JSD as an abbreviation for Jensen–Shannon Distance. In the mathematical passages in this section, JSD specifically refers to the Jensen–Shannon Divergence. The Jensen–Shannon Distance is a symmetric measure of similarity between two probability distributions. It is based on the Kullback–Leibler (KL) divergence.

Let $P={\left\{P\left(x\right)\right\}}_{x\in X}$ and $Q={\left\{Q\left(x\right)\right\}}_{x\in X}$ be two discrete probability distributions defined over the same probability space X.

The Kullback–Leibler divergence from *Q* to *P* is defined as follows:

(A6) $$D_{KL}\left(P||Q\right)=\sum_{x\in X}P\left(x\right)\log\frac{P(x)}{Q(x)}.$$

where the logarithm is typically the natural logarithm (ln) or base-2 logarithm ($\log_{2}$). We adopt the conventions $0\log\frac{0}{q}=0$ and $p\log\frac{p}{0}=\infty$ for p>0. For JSD to be finite, *P* must be absolutely continuous with respect to *Q* (i.e., if Q(x)=0 then P(x)=0).

Let $M=\frac{1}{2}\left(P+Q\right)$ be the average distribution, i.e., $M\left(x\right)=\frac{1}{2}\left(P\left(x\right)+Q\left(x\right)\right)$ for all x∈X.

The Jensen–Shannon Divergence between *P* and *Q* is then defined as follows:

(A7) $$JSD\left(P||Q\right)=\frac{1}{2}D_{KL}\left(P||M\right)+\frac{1}{2}D_{KL}\left(Q||M\right).$$

The Jensen–Shannon Distance is the square root of the Jensen–Shannon Divergence:

(A8) $$\mathrm{JS}\;\mathrm{Distance}\left(P,Q\right)=\sqrt{JSD(P||Q)}.$$

**Properties:**

- JSD is symmetric: JSD(P||Q)=JSD(Q||P).
- JSD is non-negative: JSD(P||Q)≥0.
- JSD is bounded: 0≤JSD(P||Q)≤ln2 (if using natural log) or 0≤JSD(P||Q)≤1 (if using $\log_{2}$).
- JSD(P||Q)=0 if and only if P=Q.
- The JS Distance (square root of JSD) is a true metric.

### Appendix E.3. Cramér’s V

Cramér’s V is a measure of association between two nominal categorical variables. It is based on the chi-squared ($\chi^{2}$) statistic.

Consider a contingency table with *r* rows and *c* columns, where $O_{ij}$ is the observed frequency in the cell at row *i* and column *j*. Let *N* be the total number of observations ($N=\sum_{i=1}^{r}\sum_{j=1}^{c}O_{ij}$).

The expected frequency $E_{ij}$ for cell (i,j) under the null hypothesis of independence is as follows:

(A9) $$E_{ij}=\frac{\left(\sum_{k=1}^{c}O_{ik}\right)\left(\sum_{l=1}^{r}O_{lj}\right)}{N}=\frac{R_{i}C_{j}}{N}.$$

where $R_{i}=\sum_{k=1}^{c}O_{ik}$ is the total for row *i*, and $C_{j}=\sum_{l=1}^{r}O_{lj}$ is the total for column *j*.

The chi-squared statistic is calculated as follows:

(A10) $$\chi^{2}=\sum_{i=1}^{r}\sum_{j=1}^{c}\frac{{\left(O_{ij}-E_{ij}\right)}^{2}}{E_{ij}}.$$

Cramér’s V is then defined as follows:

(A11) $$V=\sqrt{\frac{\chi^{2}}{N\cdot \min(r-1,c-1)}}.$$

**Properties:**

- *V* ranges from 0 to 1, inclusive.
- V=0 indicates no association between the variables.
- V=1 indicates a perfect association between the variables.
- It is a symmetric measure.

While interpretation can depend on the field of study and the specific context, the following are common (though not universal) guidelines for the strength of association indicated by Cramér’s V values for tables larger than 2 × 2 (for 2 × 2 tables, *V* is equivalent to the phi coefficient, and similar interpretations apply):

- 0.00 to < 0.10: Negligible or very weak association.
- 0.10 to < 0.20 (or < 0.30): Weak or small association.
- 0.20 (or 0.30) to < 0.40 (or < 0.50): Moderate association.
- 0.40 (or 0.50) and above: Strong or large association.

The value of $df^{*}=\min\left(r-1,c-1\right)$ also influences interpretation, with lower $df^{*}$ values sometimes having slightly different thresholds for “strong” (e.g., for $df^{*}=1$, a *V* of 0.10 is small, 0.30 medium, 0.50 large, following Cohen’s conventions for effect sizes). It is always advisable to consider these values in conjunction with the $\chi^{2}$ test’s *p*-value and the practical significance of the findings.
